# Production of Enriched *Sporidiobolus* sp. Yeast Biomass Cultivated on Mixed Coffee Hydrolyzate and Fat/Oil Waste Materials

**DOI:** 10.3390/microorganisms9091848

**Published:** 2021-08-31

**Authors:** Martin Szotkowski, Jiří Holub, Samuel Šimanský, Klára Hubačová, Dagmar Hladká, Andrea Němcová, Ivana Marová

**Affiliations:** Faculty of Chemistry, Brno University of Technology, Purkyňova 464/118, 612 00 Brno, Czech Republic; Jiri.Holub2@vut.cz (J.H.); xcsimansky@vutbr.cz (S.Š.); xchubacova@vutbr.cz (K.H.); Dagmar.Hladka@vut.cz (D.H.); andrea.nemcova@fch.vut.cz (A.N.); marova@fch.vut.cz (I.M.)

**Keywords:** carotenogenic yeasts, lipids, carotenoids, spent coffee grounds hydrolysate, waste animal fat, waste frying oil, coffee oil

## Abstract

One of the most addressed topics today is the transfer from a linear model of economics to a model of circular economics. It is a discipline that seeks to eliminate waste produced by various industries. The food industry generates huge amounts of waste worldwide, particularly the coffee industry, and related industries produce millions of tons of waste a year. These wastes have potential utility in biotechnology, and in the production of energy, fuels, fertilizers and nutrients, using green techniques such as anaerobic digestion, co-digestion, composting, enzymatic action, and ultrasonic and hydrothermal carbonization. This work is focused on the biotechnological use of processed spent coffee grounds (SCG) and waste fat/oil materials by some *Sporidiobolus* sp. carotenogenic yeasts in the model of circular economics. The results show that selected yeast strains are able to grow on SCG hydrolysate and are resistant to antimicrobial compounds present in media. The most productive strain *Sporidiobolus pararoseus* CCY19-9-6 was chosen for bioreactor cultivation in media with a mixture of coffee lignocellulose fraction and some fat wastes. *Sporidiobolus pararoseus* CCY19-9-6 was able to produce more than 22 g/L of biomass in mixture of SCG hydrolysate and both coffee oil and frying oil. The combined waste substrates induced the production of lipidic metabolites, whereby the production of carotenoids exceeded 5 mg/g of dry biomass. On media with coffee oil, this strain produced high amounts of ubiquinone (8.265 ± 1.648 mg/g) and ergosterol (13.485 ± 1.275 mg/g). Overall, the results prove that a combination of waste substrates is a promising option for the production of carotenoid- and lipid-enriched yeast biomass.

## 1. Introduction 

Today, it is important to pay attention to the carbon footprint of humanity in the environment; for this reason, it is necessary to focus on the quality of waste material management and their re-integration into the circular economy in the form of usable waste substrates [1,2]. Such wastes have a significant impact on the quality of the environment, as there is an over-generation of greenhouse gases, an accumulation of waste in landfills and in water, land occupation, and overall environmental pollution. For example, the chemical industry uses about 90% of fossil resources (oil, etc.) and other raw energy materials. A suitable alternative for the chemical industry would be the use of mixtures of carbon sources, both conventional (starch, molasses, etc.) and unconventional raw materials (CO_2_, methane, glycerol, agro- and municipal waste, etc.), including the involvement of biotechnological steps. The food and agricultural industries generate millions of tons of waste materials per year, which can be further recovered. The development in the field of biotechnology has created a new way to use these materials as cheap substrates for the growth of microorganisms. 

The coffee industry is a regularly growing industry that produces more coffee beans and associated products, such as instant coffee, every year. A by-product of coffee production is spent coffee grounds (SCG), which are produced in millions of tons every year [3,4,5], and are used, for example, to generate the heat necessary for production processes, or are composted [6,7]. During these processes, there is a massive release of CO_2_ into the air, which increases the carbon footprint in the environment. SCG is a very rich source of complex carbon compounds, mainly of a carbohydrate and lipid nature, which, via appropriate processing, provide a suitable source of nutrients for microorganisms [8,9]. Coffee oil obtained by SCG extraction is rich in PUFA and MUFA, and is considered a suitable high-energy carbon source [9].

A cheaper and easier-to-obtain waste lipid substrate is waste frying oil (WFO). WFO is also rich in monounsaturated fatty acids (MUFA) and polyunsaturated fatty acids (PUFA), but contains substances produced by its burning during frying [10,11]. WFO is formed during the production of fried foods in restaurants and pre-fried food factories. The annual world production of waste frying oil is 0.9–1.5 million tons [10,12]. WFOs are standardly used in biogas plants for biogas production or for the production of biodiesel [13,14], but these products are also burned and, as a result, as in the case of SCG, they also increase the carbon load on the environment [15,16].

Carotenogenic yeasts are oleogenic microorganisms known for their characteristic red color, which is derived from the carotenoids produced by yeasts themselves. These yeasts are capable of utilizing a large variety of different carbon sources (fats, oils, glycerol) and accumulating large amounts of lipids in the biomass (up to 80%) [17,18]. They are ubiquitous microorganisms found in all habitats. There is a possibility of utilizing the above-mentioned wastes to produce lipid-, carotenoid- and β-glucan-enriched biomass [19,20]. To fully utilize SCG, it is necessary to pre-treat this complex material, because yeasts do not have cellulase enzymes. Previous research has shown that SCG is a suitable source of nutrients for the cultivation of microorganisms [8]. SCG hydrolysate promotes high yeast biomass production. On the other hand, the whole process of SCG hydrolysate preparation is complex and expensive. 

In this work, we propose the use of combined waste substrates; first, a simple carbon source promotes high and fast biomass production, and second, a complex source induces the production of stress response metabolites (carotenoids, lipids). Thus, the obstacle to using the costly sugar substrate (SCG hydrolysate) are minimized by the use of a second untreated substrate, which further lowers the overall costs of cultivation. This approach also significantly extends the production time of microorganisms and exerts higher stress levels on carotenogenic yeasts.

## 2. Materials and Methods

### 2.1. Yeast Strains

Based on our preliminary results [19], several yeast strains were enrolled in this study, as follows: *Sporidiobolus roseus* CCY 19-6-4, *Sporidiobolus pararoseus* CCY 19-9-6, *Sporidiobolus metaroseus* CCY 19-6-20, *Sporidiobolus salmonicolor* CCY 19-4-6 and *Rhodosporidium toruloides* CCY 062-002-004. All strains were purchased from Culture Collection of Yeasts (CCY; Institute of Chemistry, Slovak Academy of Sciences, Bratislava, Slovak Republic), and preserved in cryovials (YPD media with 50% glycerol solution) in −82 °C.

### 2.2. Yeast Cultivation, Hydrolysate and Media Preparation

#### 2.2.1. Yeast Inoculation

YPD media was used for all inoculation steps. For proper propagation, cultures from cryovials were inoculated on Petri dishes with YPD agar and kept at the laboratory temperature for 96 h. After sufficient proliferation, using an inoculation loop, the yeasts were inoculated into 50 mL of liquid YPD medium in 250 mL Erlenmeyer flasks with a ratio of 1 loop per 10 mL of media. After 24 h, 25 mL of culture from Inoculum I was transferred to a 500 mL Erlenmeyer flask with 125 mL of YPD media (Inoculum II). After 24 h, the culture was inoculated into the production media. All cultivations were performed at room temperature (22 °C) under constant illumination with 200 µmol·m^−2^·s^−1^ of photons on a reciprocal shaker operating at 110 rpm. The composition of the YPD media was as follows: 20 g glucose, 20 g peptone, 10 g yeast extract and 1000 mL of water. Two-stage cultivation in nutrient-rich liquid inoculation media (Inoculum I and II) was used to produce a sufficient amount of yeast cells for the next step—cultivation in production media.

#### 2.2.2. Yeast Production Media

The experimental scheme was divided into two parts. The first part was focused on testing the compatibility of the prepared spent coffee ground hydrolysate as a main carbon source. The second part of the experiment focused on the bioreactor cultivation of yeasts on combined waste substrates. The best yeast strain was cultivated on a combination of simple sugar carbon source and untreated complex lipid source (animal fat, coffee oil, waste frying oil). 

The studied yeast strains of the genera *Sporidiobolus* were cultivated on a basic mineral medium with the following composition: 4 g KH_2_PO_4_, 0.696 g MgSO_4_·7H_2_O, 1.81 g urea, 4 g (NH_4_)_2_SO_4_; 30 g glucose and 1000 mL of water. The above-mentioned media composition corresponds to a C/N ratio of 13. Glucose-based media were used as a control. Only one nitrogen source was used at a time. The yeasts were cultivated on three C/N ratios—13, 25 and 50—and two artificial nitrogen sources, where the amount of nitrogen was fixed and the carbon source concentration was changing. Yeasts were cultivated in 250 mL Erlenmeyer flasks with 60 mL of media (50 mL production media + 10 mL Inoculum II). Cultivations took place on reciprocal shakers with a shaking amplitude of 110 rpm, under constant illumination and at laboratory temperature (22 °C) for 96 h. All laboratory cultivations were performed in triplicate.

#### 2.2.3. Spent Coffee Grounds Hydrolysate Preparation

Spent coffee grounds (SCG) were obtained from a commercial coffee machine using the coffee brand Bellarom Espresso (Arabica:Robusta mixture) (LIDL, Neckarsulm, Germany). To prepare the SCG hydrolysate, the collected SCG were dried for 24 h at 80 °C. The dried SCG were then milled in a laboratory grinder to obtain particles with a size of 100–500 µm. In total, 50 g of milled SCG was extracted with 200 mL of hexane:IPA 50:50 in the Soxhlet apparatus for 60 min. Solvent residues in SCG were removed in a dryer. Then, 400 g of defatted SCG were mixed with 1200 mL of 1% sulfuric acid solution in a 2 L Pyrex bottle and then treated for 90 min at 130 °C. After acid hydrolysis, the SCG hydrolysate pH was adjusted with 5% KOH solution to 5.5–6.0 for further enzymatic processing. Then, 200 µL of Cellulase enzyme blend (Novozymes, Bagsværd, Denmark) was added, and so was the SCG hydrolysate. The mixture was heated to 45 °C. After 24 h, enzymatic hydrolysis was stopped and the SCG hydrolysate was filtered. The supernatant (SCG hydrolysate) was collected and kept at 4–8 °C for further use. The SCG hydrolysate analysis is described in Section 2.3.1.

#### 2.2.4. Waste Lipid Materials

The coffee oil was obtained during the SCG hydrolysis as a byproduct via the Soxhlet extraction of spent coffee grounds. Coffee oil extract in an IPA:Hexane 50:50 solution was evaporated under vacuum. Waste frying oil (sunflower frying oil) was obtained from a household kitchen and was filtered before use under vacuum through filtration paper to remove any leftover food particles. Waste animal fat was obtained from Norillia, Norway; its composition is described in our previous work [19]. All waste lipid materials were used in their natural form, and were not subjected to any kind of hydrolysis. Lipid wastes were stored at 4–8 °C for further use.

#### 2.2.5. Large-Scale Bioreactor Co-Cultivation

The second part of the experiment was performed in a laboratory 3.0 L bioreactor under conditions based on previous data [19]. The yeast *Sporidiobolus pararoseus* CCY 19-9-6 was chosen as the best strain for bioreactor cultivation. To compare the productivity of this strain with other red yeast genera, strain *Rhodosporidium toruloides* CCY 062-00-004 was cultivated in a bioreactor under the same conditions. For bioreactor cultivation, a C/N ratio of 25 was chosen, with urea as the main carbon source. Based on the results from our previous work [19], a combination of two carbon sources was chosen as a substrate for cultivation. SCG hydrolysate served as a fast and easy way to process the carbon source in combination with a second complex source, which was an untreated waste lipid source. The ratio between carbon sources was 25:75% SCG hydrolysate:waste oil.

Before cultivation, the SCG hydrolysate was diluted with tap water to match the carbon source concentration and poured into a reactor vessel. Waste lipids were added to the medium together with salts. After that, the bioreactor was equipped with a pre-calibrated pH probe, an oxygen electrode, an aeration ring, a stirrer, a 0.2 µm pore size filter for aeration, and a temperature sensor. Subsequently, the fermenter and the equipment were sterilized in an autoclave and then cooled to room temperature.

The bioreactor vessel was equipped with two bottles that contained 5% KOH and 5% H_2_SO_4_ and were sterilized together in an autoclave at 121 °C for 15 min. After cooling, the medium’s pH was adjusted to 6.5 and the temperature to 25 °C with gentle stirring at 80 rpm. Under these conditions, the calibration of the oxygen probe was performed. A zero pO_2_ value was set immediately after the pH and temperature were set, and a 100% pO_2_ value was set after vigorous stirring and aeration. The yeasts were inoculated into a fermenter with a ratio of 1:5. Before inoculation, the yeast culture was supplemented with antifoam agent. Cultivations always lasted 4 days, i.e., 96 h. The process parameters used during cultivation are listed in Table 1. All bioreactor cultivations were done in duplicate.

### 2.3. Analytical Methods

#### 2.3.1. SCG Hydrolysate Analysis

The prepared SCG hydrolysate was analyzed for total fermentable sugar composition, organic nitrogen (protein, peptides) content and total phenolic content. For total fermentable sugars analysis, 200 µL of SCG hydrolysate was diluted with 800 µL of MiliQ water and filtered through a 0.45 µm Nylon filter into the vial. The prepared sample was analyzed using a Dionex UltiMate 3000 series HPLC with an RI detector (Thermo Fischer Scientific, Waltham, MA, USA) on a Luna Omega Sugar column 250 mm × 4.6 mm × 2.6 µm (Phenomenex, Washington, DC, USA) using isocratic elution with mobile phase ACN: H_2_O 75:25 at a flowrate of 1.0 mL/min and temperature of 35 °C. Total sugar content was identified and evaluated using a commercial carbohydrates kit (Merck, Burlington, MA, USA).

For total phenolic content, a sample of SCG hydrolysate was diluted with MiliQ water and filtered through a 0.45 µm Nylon filter into the vial. Samples were measured on a Dionex Ultimate series HPLC with a Vanquish DAD detector (Thermo Fischer Scientific, Waltham, MA, USA) on a Kinetex F5 column 150 mm × 4.6 mm × 2.6 µm (Phenomenex, Washington, DC, USA) with a flowrate of 0.4 mL/min using the gradient elution described in Table 2. Separation was performed at 35 °C. Phenolic compounds were identified using commercial standards (Merck, Burlington, MA, USA). Chromatographic data were evaluated using Chromeleon 7.2 software. Total phenolic content was calculated as a sum of all of these.

#### 2.3.2. Cell Dry Weight

Samples taken from cultivation media (20 mL) were centrifuged at 6000 rpm for 3 min. The supernatant was collected for further analyses (pH) and stored at −20 °C. The yeast cells were then washed twice with the mixture of distilled water and hexane 1:1 (*v*/*v*) and suspended in 1 mL of distilled water. Then, purified biomass was quantitatively transferred into Eppendorf tubes, frozen at −82 °C and then freeze-dried. After determining their weight, to calculate CDW, dried cells were used for the analysis of lipid-soluble metabolites-carotenoids, ergosterol, ubiquinone and lipids.

#### 2.3.3. Lipid Metabolite Analysis

The total carotenoid, sterols and coenzyme Q contents were determined using the HPLC/PDA method. Samples of freeze-dried yeast biomass were properly mixed and weighed (approx. 15–25 mg) and rehydrated with 1 mL of MiliQ water for 30 min. Excess water was removed by centrifugation at 12,000 rpm, and 1 mL of methanol and about 0.5 mL of glass beads (0.2–0.5 mm diameter) were added to the sample. The sample was vortexed for 20 min, then transferred to a 15 mL tube and washed with 2 mL of chloroform. The mixture was further vortexed for 10 min. Then, 1 mL of water was added, and the tube was allowed to stabilize for two phases after shaking. The lower chloroform phase was quantitatively transferred to a clean tube and dried under an inert nitrogen atmosphere. The dried sample was dissolved in 1 mL of mixture EtAc:ACN (2:1) and filtered through a 0.45 μm PTFE filter into the vial. Samples were measured on a Dionex Ultimate series HPLC with a Vanquish DAD detector (Thermo Fischer Scientific, Waltham, MA, USA) on a Kinetex C18-EVO column 150 mm × 4.6 mm × 5 µm (Phenomenex, Washington, DC, USA) using gradient separation with mobile phase A (ACN: MeOH: Tris HCl pH = 8; 84:2:14) and mobile phase B (MeOH: EtAc; 60:40) at a flowrate of 1.2 mL/min and 25 °C. The gradient program is listed in Table 3 [20].

Carotenoid pigments were detected at 445 nm. Chromatographic data were evaluated using Chromeleon 7.2. software. Total carotenoid, sterol and coenzyme production were identified and evaluated using commercial standards (Merck, Burlington, MA, USA) and external calibration, as in [20]. 

#### 2.3.4. Lipids and Fatty Acids

Total lipids and individual fatty acids were determined by optimized GC/FID analysis. Approx. 10–15 mg of freeze-dried yeast biomass was put into a 2.0 mL crimp neck vial together with 1.8 mL 15% (*v*/*v*) H_2_SO_4_ in methanol, capped with an aluminum cap and heated at 85 °C for 2 h. After the transesterification process, the mixture was transferred quantitatively into a 5 mL vial and neutralized with 0.5 mL of 0.005 M NaOH. The FAMEs were converted to the non-polar phase by adding 1 mL of n-hexane and shaking vigorously. The total lipids and fatty acids profile were determined by gas chromatography/flame ionization detection (GC/FID) analysis. The GC analysis of FAMEs was carried out on a TRACETM 1300 Gas Chromatograph (Thermo Fischer Scientific, USA) equipped with a flame ionization detector, an Al 1310 autosampler and a Zebron ZB-FAME column (30 m, 0.25 mm, 0.20 μm) (Phenomenex, Washington, DC, USA). The temperature program is listed in Table 4. Individual FAMEs were identified using commercial standard Supelco 37 Component FAME Mix (Sigma Aldrich, SRN, St. Louis, MO, USA). The internal standard method was used for quantification via the addition of 0.5 mg/mL of heptadecanoic acid (Sigma Aldrich, SRN, St. Louis, MO, USA) into the transesterification mixture. Chromatography data were evaluated using Chromeleon software 7.2 [19].

### 2.4. Statistical Analysis

The growth experiments in Erlenmeyer flasks were carried out in triplicate, and bioreactor cultivations in duplicate. The presented results are the means of the replicates, and the standard deviations are shown as error bars in the figures. Data handling and statistics were performed using the Excel software package (Microsoft Excel 2013, Microsoft Corp., Redmond, WA, USA).

## 3. Results 

### 3.1. Screening Cultivation of the Yeasts of Sporidiobolus Genera on SCG Hydrolysate

The experimental part started with the screening cultivation of *Sporidiobolus* and *Sporobolomyces* yeasts on media containing processed SCG hydrolysate in combination with the nitrogen sources ammonium sulfate and urea. In the experiment, cultures were taken at three C/N ratios—13, 25 and 50—where the amount of nitrogen source was kept constant and the concentration of source C increased.

#### 3.1.1. Cultivation on Media with C/N Ratio 13

The experiment started with cultivation at the lowest C/N ratio, which is more suitable for the production of carotenoids [19]. It was also used to test the resistance of carotenogenic yeasts to the antimicrobials present in the SCG hydrolysate. The results show that all tested members of the genus *Sporidiobolus* were able to grow to some extent on SCG hydrolysate, and are therefore resistant to phenolics and the other antimicrobials present in SCG. In terms of biomass production, the best screening results were obtained by the strains *Sporidiobolus pararoseus* CCY 19-9-6 and *Sporobolomyces salmonicolor* CCY 19-20-3 (see Table 5). In the case of both strains, as the more suitable N source, ammonium sulfate was found. Laboratory flask cultivation achieved biomass production of 10.03 ± 0.52 and 10.40 ± 0.62 g/L. Biomass production in the urea-based media was lower by 10–15% for all strains, with the exception of the *S. metaroseus* CCY 19-6-20 strain, which produced more biomass on urea-containing medium. 

**Figure 1:**
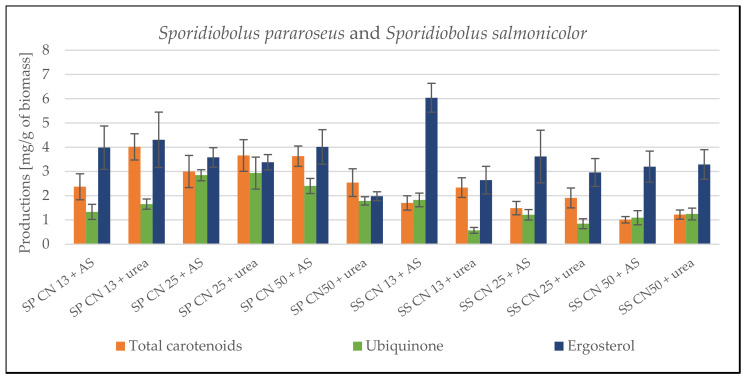

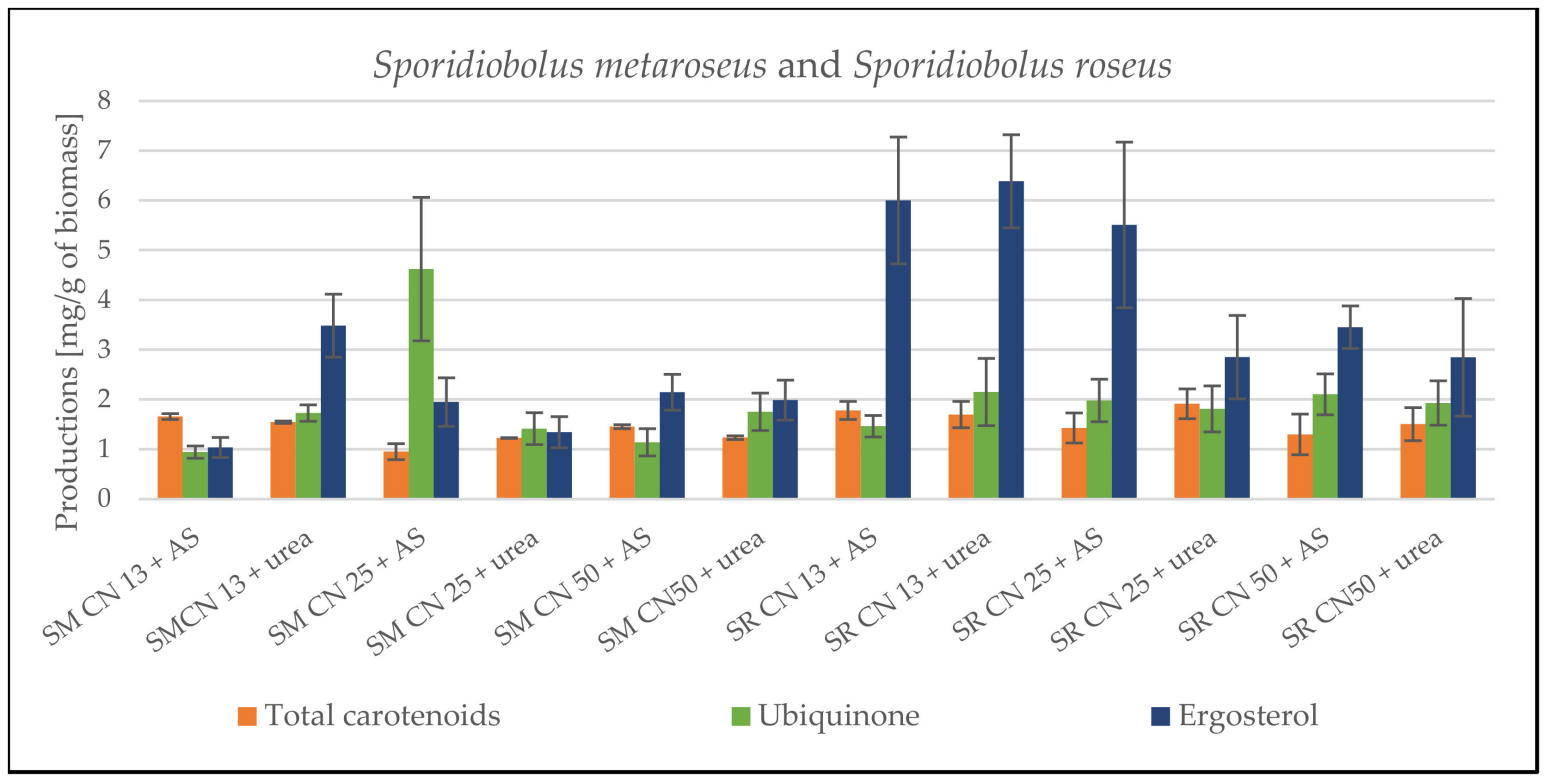
HPLC analysis results of total carotenoids, ergosterol and ubiquinone production in screening cultivation on SCG hydrolysate. Productions are listed in mg/g of cell dry weight. Abbreviations: SP—*Sporidiobolus pararoseus;* SS—*Sporidiobolus salmonicolor;* SM—*Sporidiobolus metaroseus;* SR—*Sporidiobolus roseus*.

The HPLC analysis data show the opposite trend to biomass production (Figure 1), where the addition of urea into the medium induced an increased production of lipid metabolites (pigments, ergosterol and ubiquinone). The carotenoid production was clearly dominated by the *S. pararoseus* strain, whose carotenoid yield reached 4.01 ± 0.541 mg/g of dry biomass. Compared to other strains, this is more than double the production. The results also show that the major pigments produced on urea by all studied yeasts are torulene and beta-carotene. An increase in the proportion of torularhodin is observed in the ammonium sulfate media. In terms of ergosterol production, the best producing strain was *S. roseus*, in which more than 6 mg/g of dry biomass was formed in both types of media. A Comparable production rate of 6.039 ± 0.596 mg/g was achieved by the *S. salmonicolor* strain. Regarding ubiquinone, the highest production of 2.151 ± 0.677 mg/g was achieved in this medium with the *S. roseus* strain (Figure 1). The complete HPLC results describing the content of each of the carotenoid pigments are shown in Tables S1–S4.

**Figure 2:**
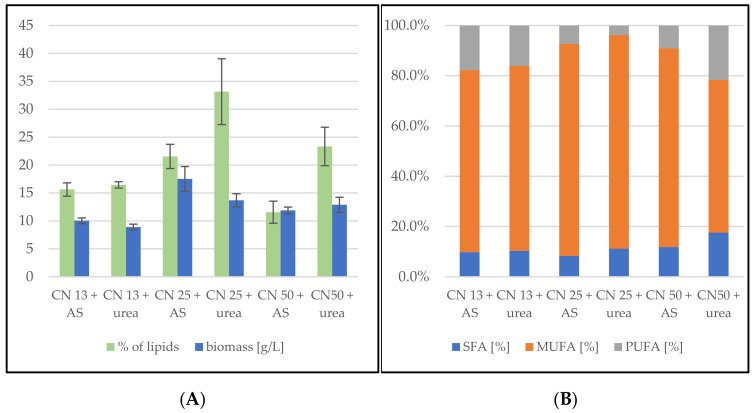
*Sporidiobolus pararoseus* screening cultivation on SCG hydrolysate media with different C/N ratios and different nitrogen source. (**A**) Biomass production (g/L) and total lipid production [%]; (**B**) fatty acid profile (%).

The lipid production data introduced in Figure 2 show a stable trend among the tested strains, with between 10 and 15% of lipids in the biomass accumulating on ammonium sulfate media. On the contrary, the urea media showed significant differences between strains. Here, the highest productions of 31.45 ± 0.92% of lipids were measured in the *S. salmonicolor* strain, and 25.49 ± 1.41% in the *S. roseus* strain, respectively. As for the fatty acid profile, a high UFA content of more than 90% was produced in all strains, with the exception of the *S. metaroseus* strain, where the UFA content was around 80%. Interestingly, in the case of the *S. pararoseus* strain, the lipids were mostly formed from monounsaturated oleic acid. In conclusion, at the C/N ratio = 13, *Sporobolomyces* sp. red yeast predominantly produces pigments and other lipid-soluble substances. Except for some strains, lipid production is low and biomass production is better. The results are shown in Figures S1–S3.

#### 3.1.2. Cultivation on Media with C/N Ratio 25

Increased carbon source content in the medium led to significant changes in the production characteristics of the yeast. The most significant changes were observed in the *S. pararoseus* strain, which produced 17.54 ± 2.21 g/L of biomass on the ammonium sulfate medium (Table 5), which was the absolute maximum of all screened cultures. Even in the case of other strains, there was an increase in biomass production on all types of media, where more than 10 g/L was achieved. An exception is the *S. metaroseus* strain, whose biomass production was lower than in the media with C/N 13. In this strain, an increased concentration of the carbon source was probably associated with the higher content of phenolic substances already leading to growth inhibition. In terms of the production of lipid metabolites, there was generally a slightly decreased or balanced production in all monitored strains. The highest carotenoid production of 3.658 ± 0.653 mg/g was achieved by the *S. pararoseus* strain on urea-containing medium (Figure 1). Carotenoid production generally showed the same trend as in previous experiments for all strains—higher production was measured in urea-based media. The profile of the carotenoids produced remained unchanged, and the major pigments produced were beta-carotene and torulene. Ergosterol and ubiquinone production were comparable or slightly reduced. The best producer of ergosterol was the *S. roseus* strain (5.508 ± 1.666 mg/g of CDW), and in the case of ubiquinone, it was the strain of *S. metaroseus*, with a maximum of 4.621 ± 1.444 mg/g of dry biomass. In both cases, the highest production was on ammonium sulfate media. The complete HPLC results describing the content of each carotenoid pigments are shown in Tables S1–S11.

The increased concentration of the carbon source led primarily to an increase in lipid content in the *S. pararoseus* strain (Figure 2). Lipid production reached 21.56 ± 2.16% on AS medium and 33.17 ± 5.90% of lipids in biomass on urea medium. The same trend was observed for the *S. metaroseus* strain, with 31.46 ± 2.52% lipids in biomass on the urea medium. Unfortunately, other strains displayed reduced lipid production. Regarding the fatty acid profile, no significant changes were observed, and the strains produced in general a similar fatty acid content compared to the C/N 13 medium (Figures S1–S3).

#### 3.1.3. Cultivation on Media with C/N Ratio 50

Screening experiments continued by cultivation at a C/N ratio of 50. All strains exhibited a decrease in biomass production compared to C/N 25 with the exception of the *S. roseus* strain, where the highest biomass production of 14.96 ± 1.08 g/L was measured (Table 5). The most probably reason is the increased concentration of phenolic substances and other antimicrobial substances in SCG hydrolysate medium, which led to growth inhibition. The higher C/N ratio had a slightly negative impact on the production of carotenoid pigments, which decreased in all studied strains except for *S. pararoseus*, in which 3.633 ± 0.420 mg/g dry biomass was measured (Figure 1). This is the highest yield of carotenoids in ammonium sulfate media in all studied strains. In terms of ubiquinone and ergosterol production, no clear trend was found in the studied strains. A reduced production of carotenoids and biomass, on the other hand, was replaced by higher lipid content in biomass. Compared to the lowest C/N ratio, an increase in the lipid content of the biomass was observed in all strains. Again, the fatty acid profile did not show significant changes. It is worth mentioning only the abrupt increase in the SFA content to 40% in the case of the *S. metaroseus* strain cultivated on urea (Figure S1).

Based on the previous screening results and our previous work with lipid substrates [19], the strain *Sporidiobolus pararoseus* CCY 19-9-6 was chosen for cultivation in the laboratory 3 L bioreactor in media with combined waste substrates.

### 3.2. Bioreactor Cultivation SCG Hydrolysate and Waste Lipid Substrates

Selected yeast strains *S. pararoseus* and *R. toruloides* were cultivated on simple mineral media containing a combination of two waste substrates: (i) SCG hydrolysate and (ii) some waste lipid substrates, in the ratio of 1:3. Urea was used as a nitrogen source. The strain *R. toruloides* was used to compare the productivity of the best *Sporidiobolus* strain to the best representative of a different clade of carotenogenic yeasts [21]. All cultivations lasted 96 h. The inoculation ratio of 1:5 was kept the same as in the screening cultivation.

#### 3.2.1. Bioreactor Cultivation in Medium with SCG Hydrolysate and Crude Waste Animal Fat

**Figure 3:**
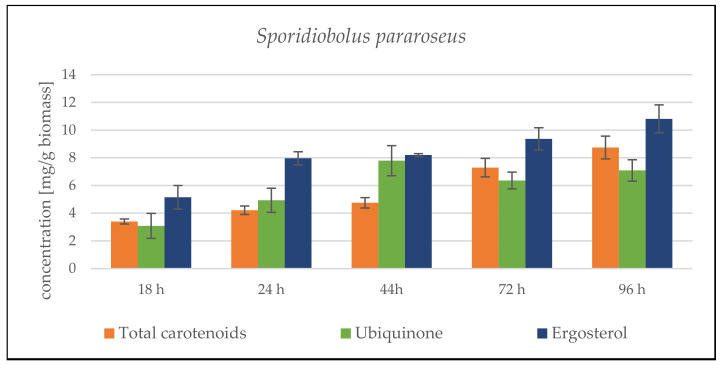

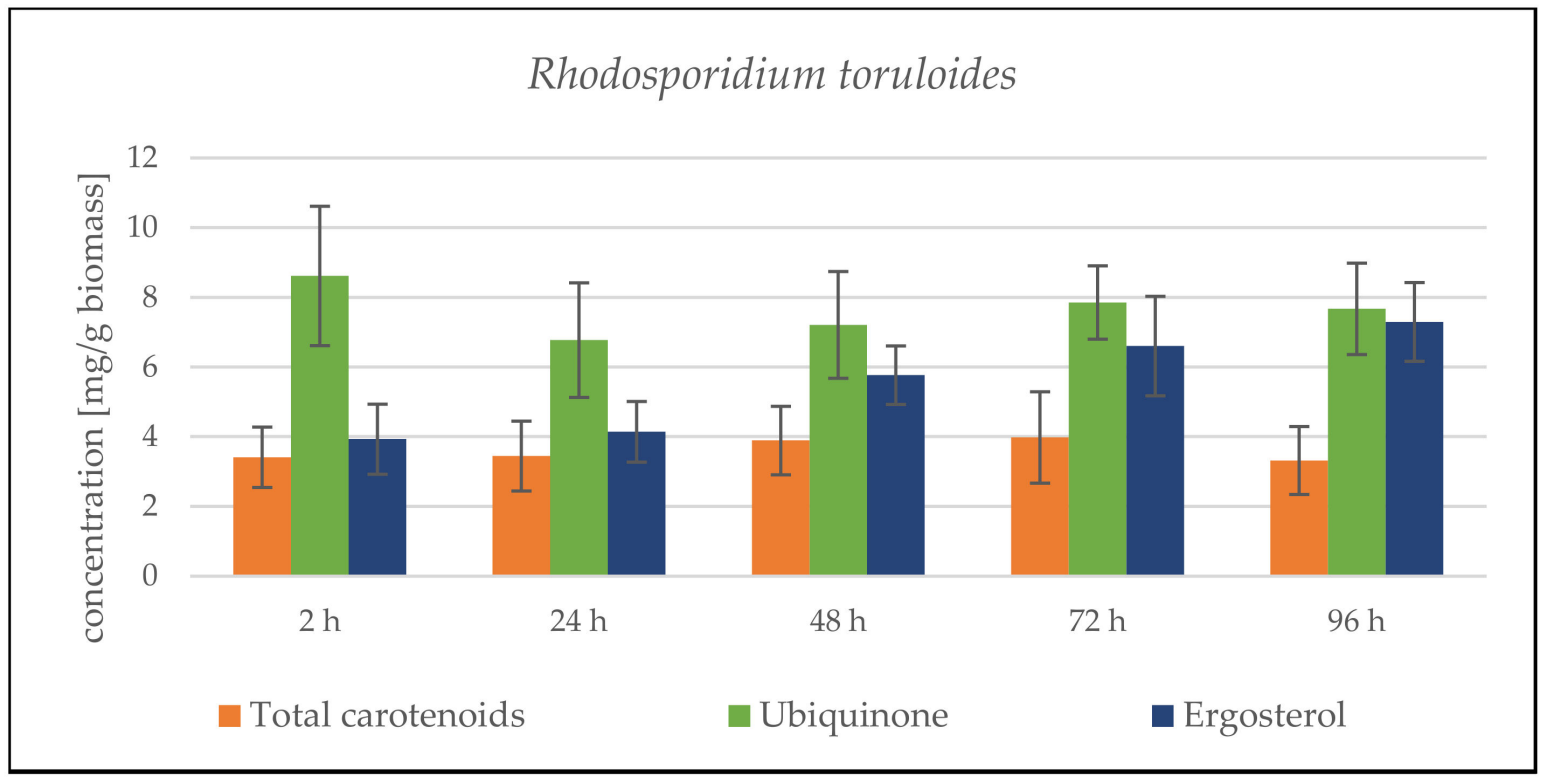
HPLC analysis of *Sporidiobolus pararoseus* and *Rhodosporidium toruloides*: 96 h bioreactor cultivation on SCG hydrolysate + waste animal fat.

The cultivation results for the combination of SCG + crude animal fat show the 4 rather average biomass productivity of both strains, compared to other lipid substrates (Figures S4 and S5). The combination of these C-sources is clearly not ideal, and the expected smooth transition of the yeast from a single sugar substrate to a second complex lipid substrate did not occur. During cultivation, there was probably a low induction of lipase production and insufficient processing of the fat substrate. Another limiting factor was the solid state of the fat under the given culture conditions (primarily cultivation temperature). The total biomass production in *S. pararoseus* was only 6.32 ± 0.78 g/L. In the *R. toruloides* strain, a better utilization of substrates and a higher biomass production of 10.75 ± 1.17 g/L were observed. The lower biomass production led to an increased production of targeted metabolites. The carotenoid production in both strains exceeded 4 mg/g, with a maximum of 8.745 ± 0.819 mg/g reached by the *S. pararoseus* strain at the end of the cultivation (Figure 3). The maximum production of the *R. toruloides* strain at 72 h was about one half of this value—3.981 ± 0.672 mg/g. In both strains, ergosterol production was induced by these substrates from the beginning and reached a maximum in the final stages of cultivation;—10.817 ± 1.012 mg/g at 96 h for *S. pararoseus* and 8.853 ± 1.308 mg/g at 78 h for *R. toruloides* (Figure 3).

Lipid production in both strains showed a decreasing trend and ranged from 15 to 20% at the end of the cultivation (Figures S4 and S5). The fatty acid profile showed the same trends as in the case of screening cultures, where the contents of unsaturated fatty acids in the biomass increased over time, which at the end of the cultivation accounted for approximately 90% of the content of all fatty acids. While in the *S. pararoseus* cells, triacylglycerols were formed by the majority of monounsaturated fatty acids, in the *R. toruloides* the distribution of MUFA and PUFA was balanced.

From the overall results, it is clear that due to the incomplete utilization of the used fat, most of the yeast biomass production properties can be attributed to the use of SCG hydrolysate, and thus, the combination of substrates needs to be further optimized to increase yields in this area. On the other hand, the slowed cell proliferation boosted the production of secondary metabolites, e.g., carotenoids and ergosterol. From a biotechnological point of view, waste animal fat appears to be a suitable substrate for the induction of the secondary metabolism of yeast and the induction of the production of carotenoids, ergosterol, and other substances.

#### 3.2.2. Bioreactor Cultivation in Medium with SCG Hydrolysate and Coffee Oil

Based on the results obtained from a previous experiment using a medium with coffee hydrolysate and fat, another similar lipid C source was tested in the next part of the study. Coffee hydrolysate rich in glucose, mannose, arabinose, and xylulose served as a good source of nutrients, which the yeast preferentially utilizes. The second carbon source here was coffee oil, which was obtained during the SCG processing. Coffee oil is a very valuable source of unsaturated fatty acids in comparison to, for example, waste animal fat. The use of coffee oil is based on the idea that yeasts will, during utilization, absorb large amounts of unsaturated fatty acids, and thus produce biomass with higher UFA content.

##### *Sporidiobolus pararoseus* Cultivation in Medium with SCG Hydrolysate and Coffee Oil

**Figure 4:**
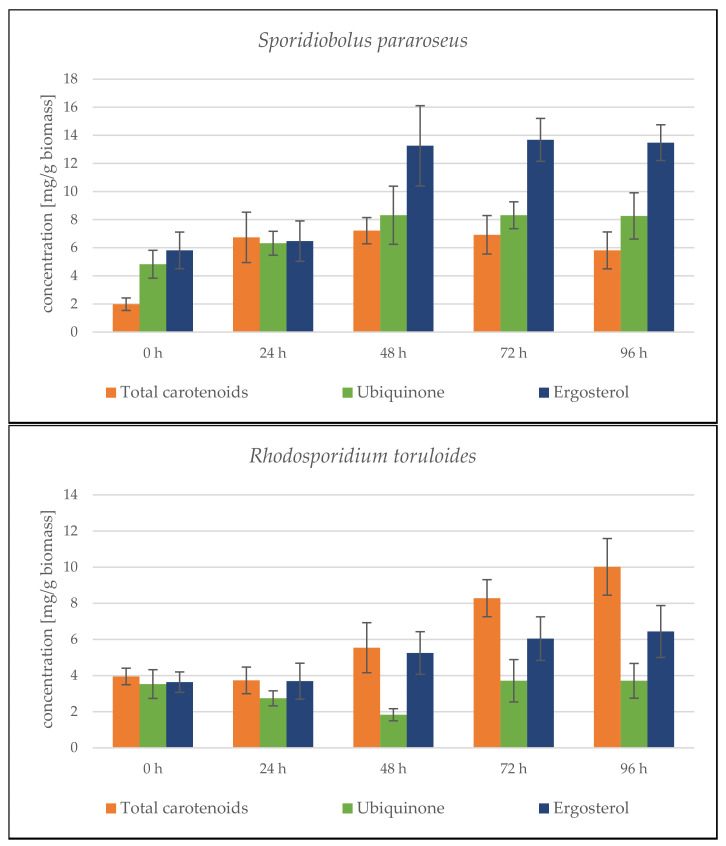
HPLC analysis of *Sporidiobolus pararoseus* and *Rhodosporidium toruloides*: 96 h bioreactor cultivation on SCG hydrolysate + coffee oil.

From the very beginning of cultivation, we observed a rapid growth of the culture. The culture quickly adapted to the SCG hydrolysate combination with coffee oil. Biomass production reached the maximum of 23.40 ± 0.88 g/L at the end of cultivation. Based on growth data, it can be assumed that if the cultivation is extended by a day or two, the biomass production will increase even further. High biomass productivity is paired with the higher production of metabolites. Carotenoid production steadily grew from the beginning of the cultivation, and reached its peak of 7.416 ± 1.179 mg/g at the 48th hour, followed by a slight decrease at the end of cultivation (Figure 4). The same trends followed ergosterol and ubiquinone production. Their production peaked at the 68th hour (13.983 ± 1.818 mg/g ergosterol). Moreover, at the 48th hour, yeast reached its maximum ubiquinone production (8.317 ± 2.067 mg/g). In the following days of cultivation, the content was stable and did not change.

**Figure 5:**
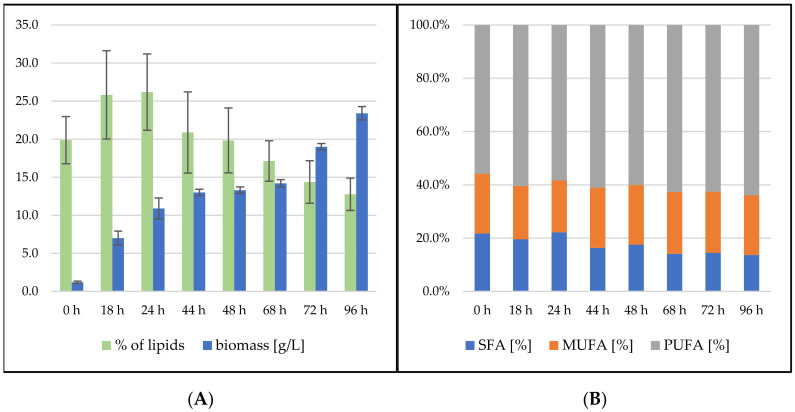
*Sporidiobolus pararoseus* bioreactor cultivation on SCG hydrolysate + coffee oil. (**A**) Biomass production (g/L) and total lipid production (%); (**B**) Fatty acid profile (%).

Lipid production data indicate differences between *S. pararoseus* and *R. toruloides*. On the first day of fermentation, we observed both the rapid growth of biomass and the accumulation of lipids in the biomass, with a maximum of 26.19 ± 5.01% of lipids at 24 h (Figure 5). A linear decrease ensued, to 12.76 ± 2.12% of lipids in the biomass, which is the lowest value of all tested conditions. The fatty acid profile of the *S. pararoseus* strain was very consistent, and the PUFA content increased at the expense of SFA, with increasing cultivation time. MUFA production was stable in the range of 22–24%. This strain showed the highest level of production of unsaturated fatty acids, making up 66.33% of lipids at the end of the cultivation.

##### *Rhodosporidium toruloides* Cultivation in Medium with SCG Hydrolysate and Coffee Oil

**Figure 6:**
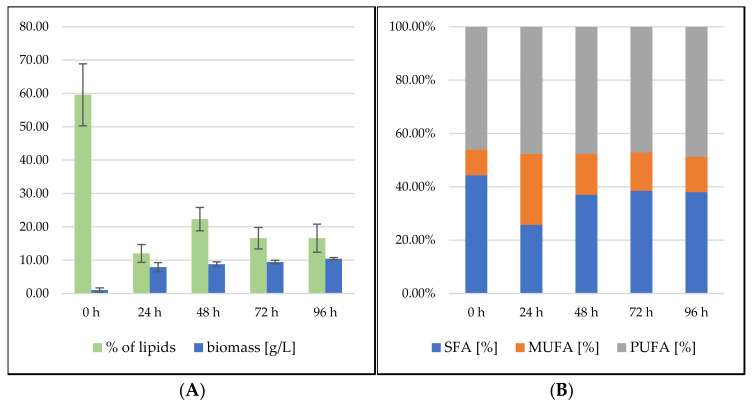
*Rhodosporidium toruloides* bioreactor cultivation on SCG hydrolysate + coffee oil. (**A**) Biomass production (g/L) and total lipid production (%); (**B**) Fatty acid profile (%).

The culture’s growth curve again showed a fast–exponential growth phase in the first two days of cultivation. In contrast to the *S. pararoseus* strain, after depleting the simple carbohydrate source, the growth slowed down, and the biomass growth reached a maximum of 10.43 ± 0.36 g/L at the 96th hour of cultivation (Figure 6). The production of lipidic metabolites steadily increased from the beginning. In the 96th hour of cultivation, carotenoid production reached 10.02 ± 1.568 mg/g, ergosterol 6.440 ± 1.433 mg/g and ubiquinone 3.710 ± 0.960 mg/g dry biomass, respectively (Figure 4). From the overall trend of the obtained data, it can be again assumed that by prolonging the cultivation time, even higher production levels of all metabolites would be achieved. The carotenoid profile is formed mainly by pigment torularhodin, which makes up more than 70% of all carotenoids.

The lipid production of *R. toruloides* strains showed a decreasing trend during the whole growth, and reached 16.58% at the end of the cultivation (Figure 6). Compared with the related genus *Sporidiobolus*, practically the same results in terms of lipid production with only slightly higher production (3%) could be seen. On the other hand, significant differences in the fatty acid profile were found, which was very stable during growth, with a fairly high SFA content of 35–40% of total fatty acids.

#### 3.2.3. Bioreactor Cultivation in Medium with SCG Hydrolysate and Waste Frying Oil

To further compare the utilization differences between solid and lipid C-sources, yeasts were cultivated under the same conditions as described in the previous two paragraphs using waste frying oil as the second lipid substrate. This experiment also provided data about the differences between coffee oil and frying oil regarding antimicrobial compounds and their effects on yeast growth.

##### *Sporidiobolus pararoseus* Cultivation in Medium with SCG Hydrolysate and Waste Frying Oil

**Figure 7:**
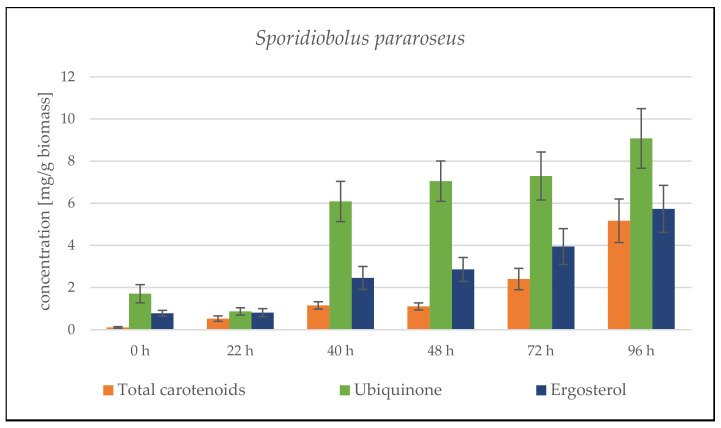

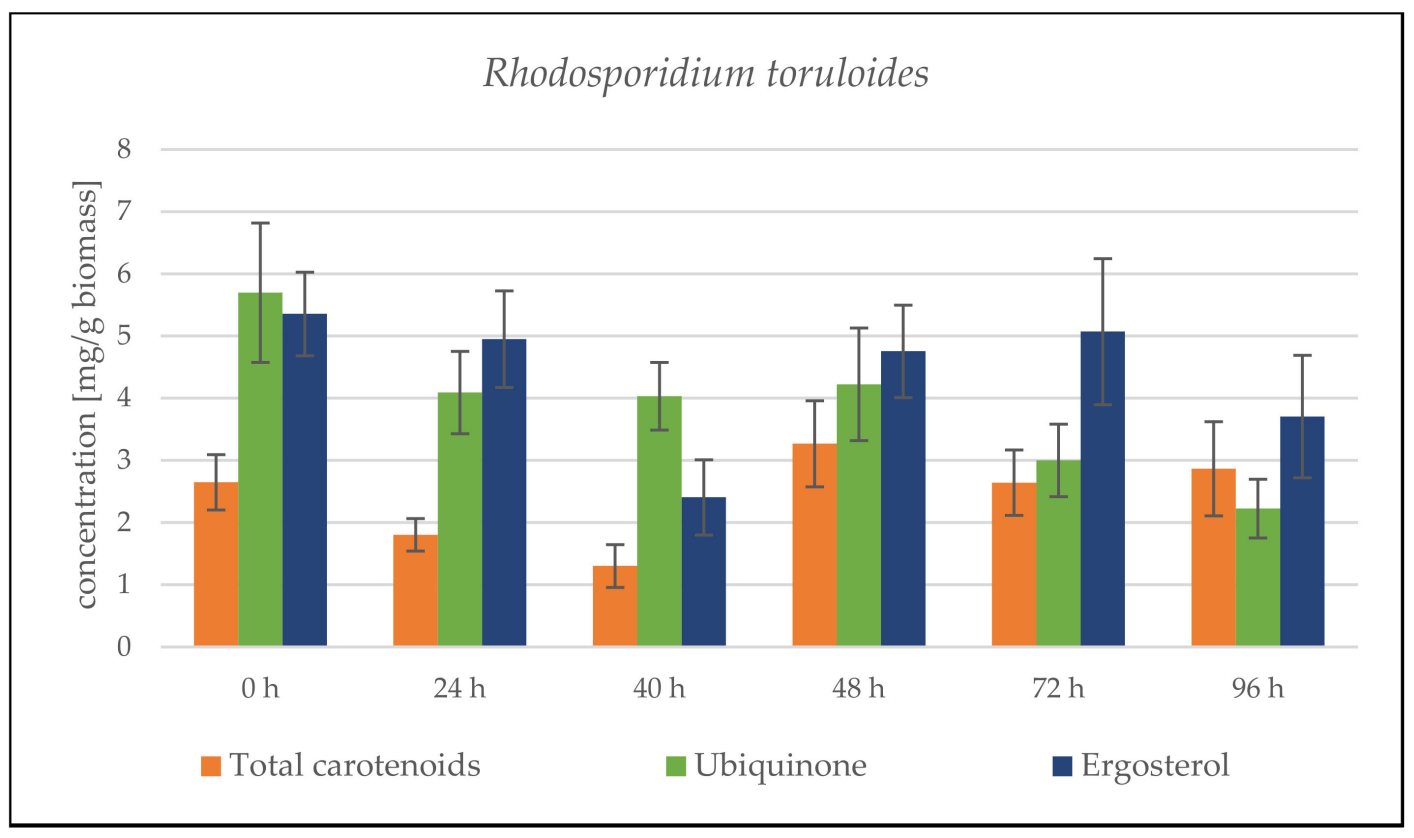
HPLC analysis of *Sporidiobolus pararoseus* and *Rhodosporidium toruloides*: 96 h bioreactor cultivation on SCG hydrolysate + waste frying oil.

Results for the *S. pararoseus* strain show similar productivity as in the media with coffee oil. The yeast achieved a very high biomass production of 21.63 ± 0.38 g/L, which is slightly lower compared to the coffee oil (23.40 ± 0.88 g/L). Additionally, under these conditions, the yeast adapted fast to the second carbon source after depleting the primary SCG hydrolysate carbon source. This phenomenon is shown in the data from the 48–60th hour of cultivation, where the yeast biomass production slowed down due to necessary metabolic shifts, and then it displayed quite stable linear growth. Again, the results show that, by prolonging the cultivation, the biomass production would continue to grow. The yeast *S. pararoseus* showed typical trends in the production of these metabolites with two production maxima that coincide with the period of transition from the metabolism of simple carbon sources to complex sources. These production properties have already been confirmed in our previous study [19]. Carotenoid production reached a maximum of 5.172 ± 1.032 mg/g in the last sample after 96 h. The highest productions of ergosterol (5.733 ± 1.115 mg/g) and ubiquinone (9.079 ± 1.417 mg/g) were also determined in the same sample (Figure 7).

**Figure 8:**
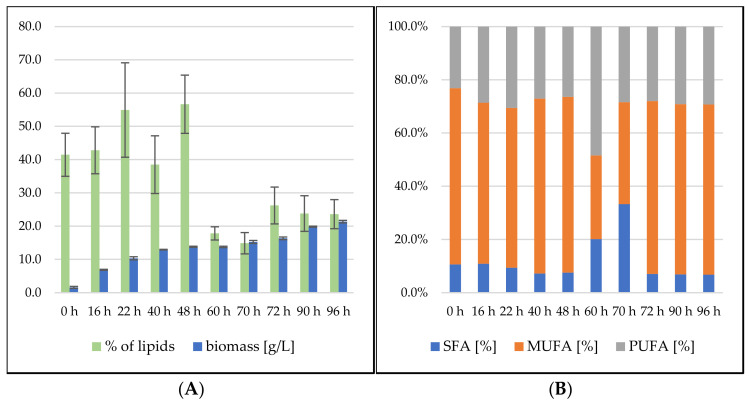
*Sporidiobolus pararoseus* bioreactor cultivation on SCG hydrolysate + waste frying oil. (**A**) Biomass production (g/L) and total lipid production (%); (**B**) Fatty acid profile (%).

The lipid production of the *S. pararoseus* strain showed very high values in the first 48 h of cultivation, during which the lipid content reached 56.65 ± 8.68% in the biomass (Figure 8). During the above-mentioned metabolic shift between the second and third days of cultivation, the lipid content dramatically dropped towards 15% and then slowly grew to 23.60 ± 4.35% to the end of cultivation. The yeast maintained a fairly stable FA profile with 85–90% of UFA during the whole cultivation, with the majority consisting of MUFA (predominantly oleic acid). The only exception was, again, the interval of metabolic shift, where SFA content reached 33.24%.

##### *Rhodosporidium toruloides* Cultivation in Medium with SCG Hydrolysate and Waste Frying Oil

**Figure 9:**
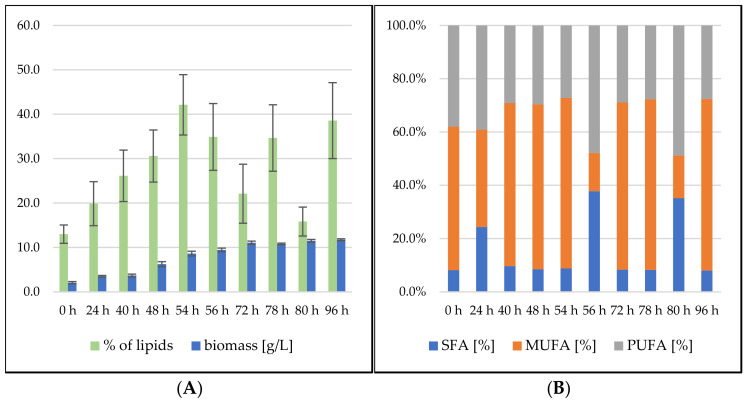
*Rhodosporidium toruloides* bioreactor cultivation on SCG hydrolysate + waste frying oil. (**A**) Biomass production (g/L) and total lipid production (%); (**B**) Fatty acid profile (%).

The yeast *R. toruloides* displayed a similar productivity on waste frying oil to coffee oil. Biomass production was quite slow, and linear growth occurred until the end, reaching a maximum of 11.75 ± 0.22 g/L (Figure 9), which is slightly more (10%) than the coffee oil media. The HPLC analysis data showed a relatively large fluctuation in the production of individual metabolites. Multiple maxima can be identified for all metabolites during cultivation, but they do not overlap in one sample. The maximum carotenoid production of 4.766 ± 0.708 mg/g was reached in the culture after 78 h (Figure 7). Here, torularhodin was produced as the major pigment. In contrast, the maximum ergosterol production of 5.791 ± 1.109 mg/g was measured at the 54th hour. The maximum ubiquinone production, 5.803 ± 1.164 mg/g of dry biomass, was reached in the culture at the 56th hour of growth.

Chromatography analysis of lipid formation and fatty acid profiles indicates the potential of this yeast for high lipid production. During cultivation, the lipid content increased to 42.12 ± 6.79% at the 54th hour, and after a decrease in the following hours, it reached two more maxima: 34.65 ± 7.47% after 78 h and 38.56 ± 8.54% after 96 h of cultivation (Figure 9). The fatty acid profile of *R. toruloides* showed a very stable production of unsaturated fatty acids, which, with a few exceptions, reached more than 90% of the total fatty acid content throughout the cultivation. From this point of view, it is possible to use this strain to produce lipids with a very high content of unsaturated fatty acids using cheap waste substrates.

## 4. Discussion

Carotenogenic yeasts are versatile microorganisms capable of growing under various conditions, whose enzymatic apparatus allows the processing of various waste substrates in the food industry and their use as part of a circular economy [1,19]. Carotenogenic yeasts such as oleogenic microorganisms are able to produce high amounts of lipids [22,23,24]. At the same time, however, they produce industrially important carotenoids [25,26], beta-glucans [20,21,22] and proteins [23]. Further, carotenogenic yeasts are able to grow simultaneously in co-culture with autotrophic microorganisms, producing enriched biomass [27]. Thus, they can be considered an ideal microorganism for the biotechnological processing of many waste materials and their valorization to high-value-added substances. Biomass enriched in this way can be used as a multifunctional complex source of vitamins and minerals in the feed industry, as food supplements, or in the pharmacy. Alternatively, individual components can be extracted and targeted for each particular market [28].

Waste substrates for the food and agricultural industries are potentially suitable sources of nutrients for biotechnological processing by microorganisms. The main advantage of these materials is twofold. The first is, of course, the price, which is lower compared to artificial sources (glucose, yeast extract). The second is the complexity of the substrate, as this material can serve as a source of multiple nutrients (C, N, S) and simultaneously as a stress factor. Using this strategy, it can be (ideally) possible to produce large amounts of microbial biomass on a cheap substrate, along with the induced production of industrially important metabolites due to induced stress.

However, in the biotechnology of carotenogenic yeasts, we encounter the problem that waste substrates used for yeast cultivation must be treated and hydrolyzed to some extent. The main reason is the low activity of the hydrolytic enzymes produced by yeast or their complete absence, especially cellulose substrates. As the substrate becomes more hydrolyzed, it becomes a more available source of nutrients for the yeast, leading to faster culture growth, but not always to higher target metabolite production [19,29]. The induction of metabolite production by yeast occurs to a higher extent in native untreated substrates. The waste material in its untreated form acts as a complex substrate, thus exerting greater pressure on the yeast, leading to the increased production of the desired metabolites [19]. It is therefore necessary to find a suitable compromise that would ensure sufficient growth of yeast biomass and, at the same time, induce the production of metabolites. In the case of animal fat and lipid substrates in general, simple glycerol can be released by hydrolysis. The disadvantage of this process is the simultaneous release of a higher concentration of free fatty acids, which, in higher concentrations, have a strong inhibitory effect on the growth and viability of yeast. In our previous work, it was confirmed that the best compromise here is partial hydrolysis [19], which releases only a enough glycerol for growth in the exponential phase, and the remaining unhydrolyzed fat induces the increased production of carotenoids and lipids.

The second option, which was studied in this work, is the combination of two different waste materials as simultaneous C-sources for some yeasts of the genus *Sporidiobolus/Sporobolomyces*. The main idea is to apply a sufficient amount of simple C-source for the sufficient growth of the biomass, and the second C-source at some excess, which is utilized by yeast after consumption of the first C-source. The second material serves not only as a complex C-source, but also induces the formation of desired metabolites. This procedure proposed by us allows the regulation of several basic parameters of the biotechnological process by modulating the amount of waste substrates used for yeast cultivation. The first is the financial complexity of the process, especially the processes necessary for the preparation of a suitable hydrolysate (use of enzymes, hydrolysis at high temperatures, filtration of the hydrolysate, etc.). The second is the induction of the production of desired metabolites.

In this work, the experiments started with the screening cultivation of selected *Sporidiobolus* strains on SCG hydrolysate prepared according to the procedure in Section 2.2.3. Yeasts were cultured at three C/N ratios with different nitrogen sources (urea and ammonium sulfate). The results confirm the ability of the carotenogenic yeasts to utilize coffee hydrolysate, and at the same time, they showed a high level of resistance to antimicrobial phenolic substances. All studied strains of the genus *Sporidiobolus* produced high amounts of biomass, with the exception of the *S. metaroseus* CCY 19-6-20 strain, whose biomass production decreased very rapidly with increasing C/N ratio, and generally did not reach the level of production of other members of the genus. The low biomass production is probably due to the higher sensitivity of this strain to inhibition by increasing concentrations of antimicrobial compounds. The production of carotenoids, ergosterol and ubiquinone in carotenogenic yeasts showed the same trend as was observed in our previous works [19], where the production of these metabolites was induced in media with a lower C/N ratio. On the SCG hydrolysate, the highest productions were measured at the lowest C/N ratio.

Based on the results of the screening part of the experiment and previous experiments, the best producing strain, *S. pararoseus* CCY 19-9-6, was cultivated in a laboratory bioreactor on a medium with a combination of two waste substrates, where the SCG hydrolysate served as the initial rapid (sugar-based) C-source needed for the rapid proliferation of cells in the exponential phase, and untreated lipid waste material was applied as the second complex C-source used by yeast in the late stage of growth. Cultivation was performed on medium with a C/N ratio of 25 and a substrate ratio 1: 3. A higher ratio of unhydrolyzed lipid substrate was chosen to reduce the potential costs of the process by increasing the content of cheaper waste, and at the same time to monitor the effect on the production of biomass and the monitored metabolites. In the experiment, three types of complex lipid substrates (animal fat, coffee oil and waste frying oil) were tested. The results of the *S. pararoseus* strain were compared with those of the *R. toruloides* CCY 062-00-004 strain, which was chosen for comparison with a previously described highly producing representative of another genus of carotenogenic yeasts [26]. The results show that the selected combination of substrates induced biomass production, and at the same time, also produced target yeast metabolites, thus confirming our assumption. 

The experiments with animal fat have highlighted the fundamental problem of this material. Under the given cultivation conditions, the fat was undissolved and remained in solid form, and, thus, it was difficult to process this nutrient using yeast cells. During cultivation, only a low utilization of this C-source was observed, and biomass production in both strains was low. On the other hand, the growth retardation of the yeast culture caused by the reduced ability to utilize this substrate led to the induction of the production of secondary metabolites and a significant increase in their total production in both strains, especially in the strain *Sporidiobolus pararoseus*. Further optimization of the cultivation conditions regarding the solid state of the waste animal fat could thus lead to even higher production levels. In the case of coffee oil or frying oil, which were in media in the liquid state, the active area for the lipases was many times higher, and, thus, a better utilization of the substrate and an increased production of biomass and metabolites occurred. The results also show the positive effects of the used waste liquid oils on the fatty acid profile. The *S. pararoseus* strain showed the significant influence of the lipid substrate on the composition of fatty acids in the final yeast biomass. When culturing the yeast in coffee oil, a significant degree of assimilation of the unsaturated fatty acids present in the coffee oil was measured, and the resulting yeast biomass contained more than 80% UFA (60% PUFA and 20% MUFA). After changing the lipid substrate, the fatty acid profile in the waste frying oil was as follows: 60–65% MUFA and 25–30% PUFA. Unsaturated fatty acids generally have a beneficial effect on human and animal health. The biomass of red yeast produced in this way, which contains a high content of unsaturated fatty acids and other valuable substances, can thus in the future serve as a dietary supplement for people, or as an animal feed [30].

The results of the study show that the proposed procedure for the cultivation of carotenogenic yeasts on a combination of two utilized waste C-sources is an effective method for obtaining a high yield of enriched yeast biomass. The selected ratio of 1:3 SCG hydrolysate and waste oil can reduce the financial costs of cultivation by replacing pure chemicals with waste substrates. At the same time, the application of untreated oil leads to a reduction in the amount of SCG in the medium and, thus, to a reduction in the costs of the preparation of a larger amount of this substrate. Furthermore, it was found that the choice of waste oil can modulate the ratio of individual groups of fatty acids in biomass. The application of a fast substrate gives the yeast enough time to synthesize the lipases needed to process the oil substrate, and further leads to a better transformation of the waste. The overall results show that even higher production levels of biomass and metabolites can be achieved by the further optimization of the process, including the ratio of both substrates, the length of cultivation, and other parameters. Moreover, the results of the present study confirm that some yeasts of the genus *Sporobolomyces* can be suitable for the industrial production of high value-added yeast biomass and metabolites under the ircular economy concept.

## 5. Conclusions

In this work, four strains of genera *Sporidiobolus* (*Sporidiobolus roseus* CCY 19-6-4, *Sporidiobolus pararoseus* CCY 19-9-6, *Sporidiobolus metaroseus* CCY 19-6-20, *Sporidiobolus salmonicolor* CCY 19-4-6) were cultivated on media based on SCG hydrolysate as the main carbon source, complex lipids as the second carbon source and two types of nitrogen sources (urea, ammonium sulfate) at different C/N ratios (13, 25, 50). The aim was first to test the yeast productivity on this waste substrate, and the yeast’s resistance to antimicrobial compounds present in the SCG hydrolysate. The results show that all tested *Sporobolomyces* strains were able to utilize the SCG hydrolysate. The highest biomass production for all strains was on the media with C/N ratio 25, which was, together with the C/N ratio 13, suitable for inducing the production of lipidic metabolites (carotenoid, ergosterol and ubiquinone). With increasing concentrations of the carbon source, the yeast’s production of lipids was improved. Thus, the highest lipid production rate was found in media with C/N 50. It can be assumed that with higher C/N ratios, the lipid content would grow further. *Sporidiobolus pararoseus* CCY 19-9-6 was selected as the best producing strain for bioreactor cultivation in media with combined waste substrates, similarly to *Rhodosporidium toruloides* CCY 062-002-004, which served as a high-producing representative of a different yeast clade for comparison. Both yeasts were cultivated on media containing SCG hydrolysate + lipid waste (crude animal fat, waste frying oil, coffee oil). The results proved our assumption that by using a combination of waste substrates, we can stimulate a higher rate of production of certain metabolites and yeast biomass. Generally, the best results were obtained with the SCG hydrolysate and liquid lipid waste materials; both strains were able to utilize both substrates. Thus, yeasts of the *Sporobolomyces* sp. can be recommended as stable and high-producing industrially applicable strains, similarly to *Rhodotorula* sp. The addition of coffee or frying oil had an interesting impact on the fatty acid profile of yeasts. Overall, the applied combination of lignocellulose and fat/oil waste materials was very effective in inducing the production of carotenoid- and lipid-enriched biomass, adhering to the conception of a circular economy. The addition of untreated oil substrate further lowers the cost of the whole biotechnology process.

## Figures and Tables

**Table 1:** Bioreactor process values during co-cultivation.

Media volume	2.0 L
Stirring	300–800 rpm, regulated by oxygen consumption
pH	6.5
pO_2_	30%
Temperature	25 °C
Aeration	3 L per minute
Illumination	300 μmol·m^2^·s^−1^ of photons

**Table 2:** Phenolic content analysis: gradient shape used during HPLC/DAD analysis.

	Retention Time (min)	Mobile Phase A (%)	Mobile Phase B (%)
1	0.0	90%	10%
2	1.0	90%	10%
3	5.0	85%	15%
4	10.0	80%	20%
5	21.0	25%	75%
6	26.0	55%	45%
7	30.0	90%	10%

**Table 3:** HPLC/DAD analysis of yeast lipid metabolites: changes in mobile phase composition during gradient elution.

	Retention Time (min)	Mobile Phase A (%)	Mobile Phase B (%)
1	0.0	100%	0%
2	13.0	0%	100%
3	19.0	0%	100%
4	20.0	100%	0%
5	25.0	100%	0%

**Table 4:** Temperature program of GC/FID analysis of FAMEs.

	Retention Time (min)	Gradient (°C·min^−1^)	Temperature (°C)	Retention (min)
1	0.000	start	-	-
2	1.000	0.000	80.000	1.000
3	5.000	15.000	140.000	0.000
4	21.667	3.000	190.000	0.000
5	24.467	25.000	260.000	1.000
6	24.467	stop	-	-

**Table 5:** Yeast biomass production in screening cultivations on SCG hydrolysate. Biomass production is listed in g/L of media.

Media Type	*S. pararoseus*	*S. metaroseus*	*S. salmonicolor*	*S. roseus*
CN 13 + AS	10.03 ± 0.52	3.41 ± 0.79	10.40 ± 0.62	7.74 ± 0.82
CN 13 + urea	8.91 ± 0.53	5.87 ± 0.37	9.44 ± 0.26	6.48 ± 0.64
CN 25 + AS	17.54 ± 2.21	3.58 ± 0.47	14.13 ± 0.49	12.09 ± 0.65
CN 25 + urea	13.69 ± 1.19	1.78 ± 0.74	12.27 ± 1.06	10.37 ± 0.69
CN 50 + AS	11.88 ± 0.62	2.65 ± 0.80	4.49 ± 0.83	11.73 ± 3.32
CN50 + urea	12.89 ± 1.34	3.021 ± 0.90	11.01 ± 1.43	14.96 ± 1.08

## Data Availability

Not applicable.

## Supplementary Materials

The supplementary materials are available online at www.mdpi.com/article/10.3390/microorganisms9091848/s1.
