# Supplementary material for: Production of Enriched Sporidiobolus sp. Yeast Biomass Cultivated on Mixed Coffee Hydrolyzate and Fat/Oil Waste Materials
Source: Microorganisms. 2021 Aug 31;9(9):1848. doi: 10.3390/microorganisms9091848 (PMC8472217; doi:10.3390/microorganisms9091848)
Supplement: Supplementary file 1 [file microorganisms-09-01848-s001.zip › microorganisms-1329340-supplementary.pdf]

microorganisms

MDPI

Suplementary material

Production of enriched *Sporidiobolus* sp. yeast biomass cultivated on mixed coffee hydrolyzate and fat/oil waste materials

Martin Szotkowski[1*], Jiri Holub[1], Samuel Simansky[1], Klára Hubacova[1], Dagmar Hladka[1], Andrea Nemcova[1] and Ivana Marova [1]

[1] Faculty of Chemistry, Brno University of Technology, Brno, Czech Republic ; xcszotkowski@fch.vut.cz
* Correspondence: xcszotkowski@fch.vut.cz; Tel.: +420 775 288 439

Contents:

**Figure S1**	*Sporidiobolus metaroseus* screening cultivation on SCG hydrolysate media with different C/N ratio and different nitrogen source.
**Figure S2**	*Sporidiobolus salmonicolor* screening cultivation on SCG hydrolysate media with different C/N ratio and different nitrogen source.
**Figure S3**	*Sporidiobolus roseus* screening cultivation on SCG hydrolysate media with different C/N ratio and different nitrogen source.
**Figure S4**	*Sporidiobolus pararoseus* cultivation on media containing SCG hydrolysate + waste animal fat. A) Biomass production [g/L] and total lipid production [%]; B) Fatty acid profile [%]
**Figure S5**	*Rhodosporidium toruloides* cultivation on media containing SCG hydrolysate + waste animal fat. A) Biomass production [g/L] and total lipid production [%]; B) Fatty acid profile [%]
**Table S1**	HPLC analysis of screening cultivations of *S. pararoseus* cultivated on media containing SCG hydrolysate with different C/N ratio. Productions are listed in mg/g of cell dry weight.
**Table S2**	HPLC analysis of screening cultivations of *S. metaroseus* cultivated on media containing SCG hydrolysate with different C/N ratio. Productions are listed in mg/g of cell dry weight.
**Table S3**	HPLC analysis of screening cultivations of *S. roseus* cultivated on media containing SCG hydrolysate with different C/N ratio. Productions are listed in mg/g of cell dry weight.
**Table S4**	HPLC analysis of screening cultivations of *S. salmonicolor* cultivated on media containing SCG hydrolysate with different C/N ratio. Productions are listed in mg/g of cell dry weight.
**Table S5**	HPLC analysis of *S. pararoseus*: 96 hours bioreactor cultivation on SCG hydrolysate + coffee oil. Productions are listed in mg/g of cell dry weight.
**Table S6**	HPLC analysis of *R. toruloides*: 96 hours bioreactor cultivation on SCG hydrolysate + coffee oil. Productions are listed in mg/g of cell dry weight
**Table S7**	HPLC analysis of *S. pararoseus*: 96 hours bioreactor cultivation on SCG hydrolysate + waste frying oil. Productions are listed in mg/g of cell dry weight.
**Table S8**	HPLC analysis of *R. toruloides*: 96 hours bioreactor cultivation on SCG hydrolysate + waste frying oil. Productions are listed in mg/g of cell dry weight.
**Table S9**	HPLC analysis of *S. pararoseus*: 96 hours bioreactor cultivation on SCG hydrolysate + waste animal fat. Productions are listed in mg/g of cell dry weight.
**Table S10**	HPLC analysis of *R. toruloides*: 96 hours bioreactor cultivation on SCG hydrolysate + waste animal fat. Productions are listed in mg/g of cell dry weight.
**Table S11**	HPLC analysis of phenolic content in different cultivation media according to the C/N ratio at the beginning of the cultivation. Productions are listed in mg/L of media.

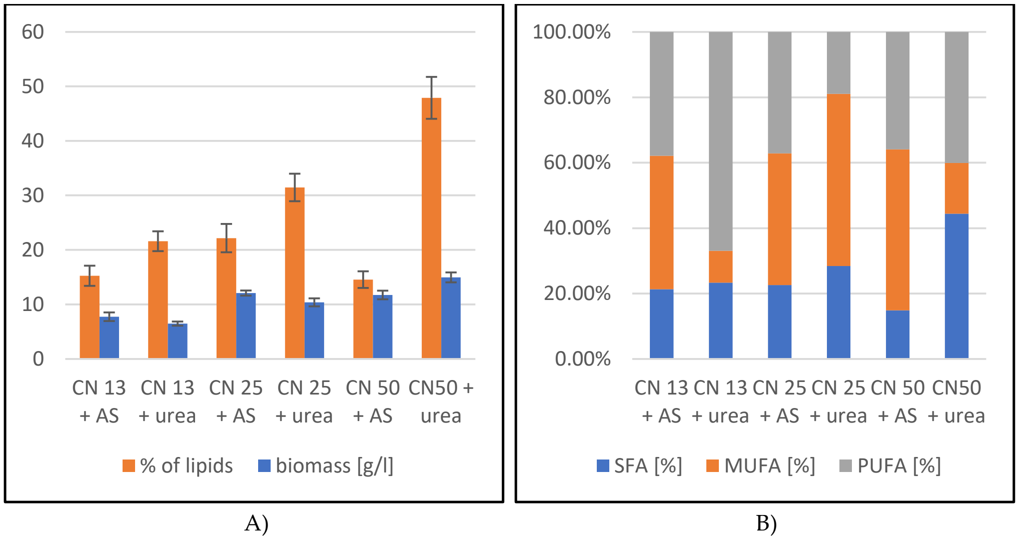

Sporidiobolus metaroseus screening cultivation on SCG hydrolysate media with different C/N ratio and different nitrogen source.
A) Biomass production [g/L] and total lipid production [%]; B) Fatty acid profile [%]

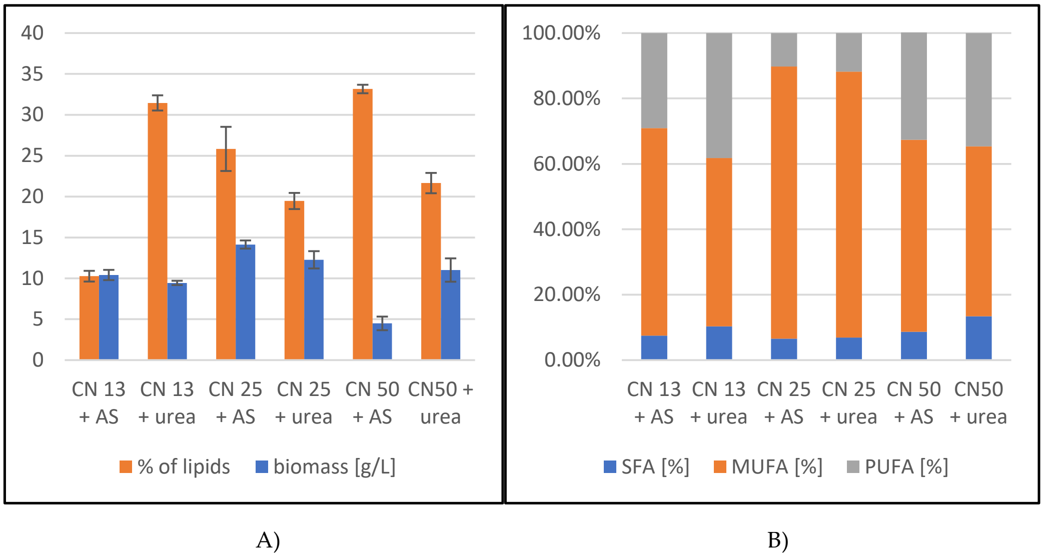

Sporidiobolus salmonicolor screening cultivation on SCG hydrolysate media with different C/N ratio and different nitrogen source.
A) Biomass production [g/L] and total lipid production [%]; B) Fatty acid profile [%]

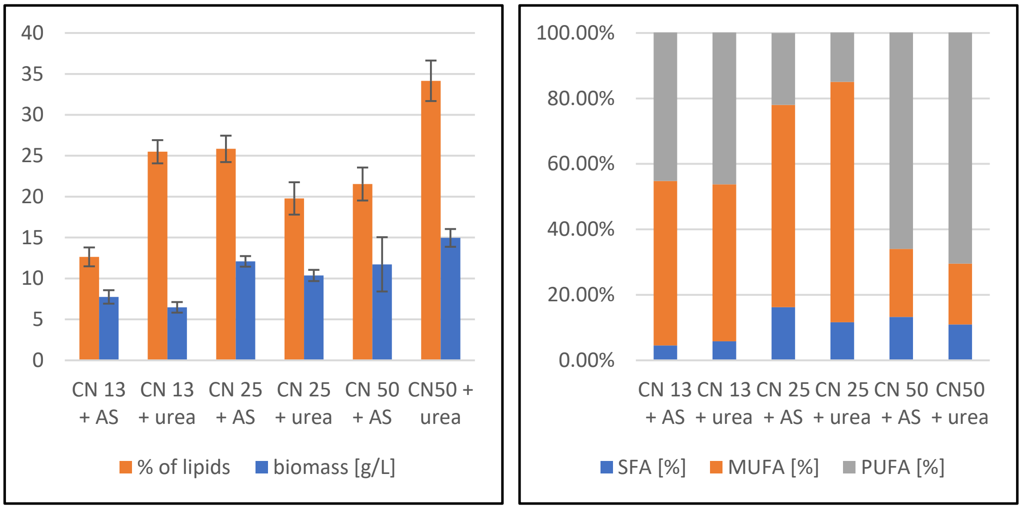

A) B)

Sporidiobolus roseus screening cultivation on SCG hydrolysate media with different C/N ratio and different nitrogen source.
A) Biomass production [g/L] and total lipid production [%]; B) Fatty acid profile [%]

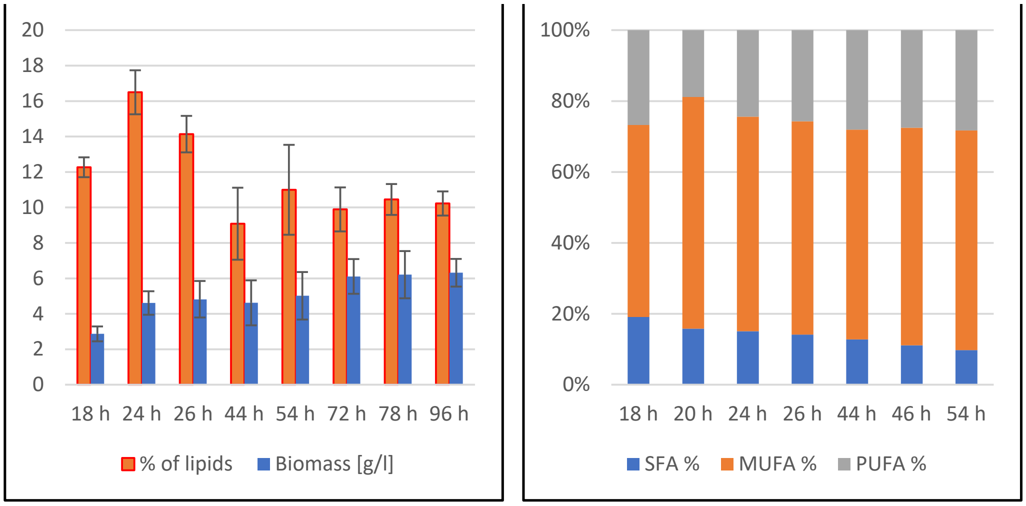

A) B)

Sporidiobolus pararoseus cultivation on media containing SCG hydrolysate + waste animal fat.
A) Biomass production [g/L] and total lipid production [%]; B) Fatty acid profile [%]

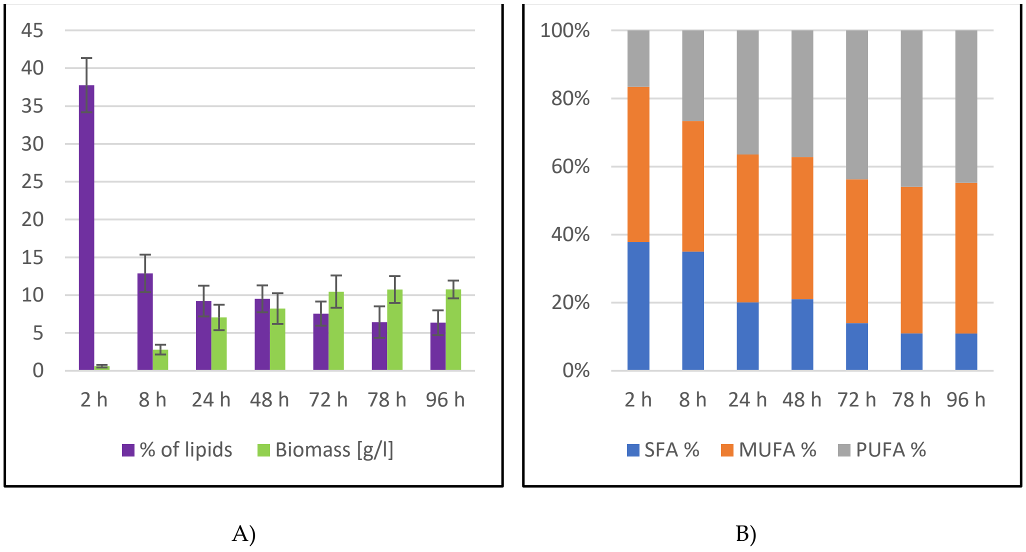

Rhodosporidium toruloides cultivation on media containing SCG hydrolysate + waste animal fat. A) Biomass production [g/L] and total lipid production [%]; B) Fatty acid profile [%]

Table S1: HPLC analysis of screening cultivations of *S. pararoseus* cultivated on media containing SCG hydrolysate with different C/N ratio. Productions are listed in mg/g of cell dry weight.

Media type	Betacarotene	Torularhodin	Torulene	Lycopene	Total carotenoids	Ubiquinone	Ergosterol
CN 13 + AS	1.222±0.153	-	0.268±0.058	0.436±0.073	2.37±0.5372	1.332±0.311	3.984±0.893
CN 13 + urea	1.191±0.197	-	1.344±0.261	0.653±0.166	4.015±0.541	1.654±0.211	4.308±1.139
CN 25 + AS	1.166±0.274	0.334±0.045	1.018±0.134	0.211±0.046	2.999±0.664	2.847±0.230	3.580±0.404
CN 25 + urea	1.648±0.332	0.319±0.063	1.518±0.220	0.014±0.003	3.658±0.653	2.934±0.659	3.381±0.317
CN 50 + AS	1.135±0.225	0.372±0.072	1.438±0.225	0.294±0.063	3.633±0.420	2.403±0.311	4.013±0.712
CN50 + urea	1.155±0.233	0.203±0.034	1.005±0.134	0.007±0.002	2.541±0.571	1.786±0.170	1.978±0.183

Table S2: HPLC analysis of screening cultivations of *S. metaroseus* cultivated on media containing SCG hydrolysate with different C/N ratio. Productions are listed in mg/g of cell dry weight.

Media type	Betacarotene	Torularhodin	Torulene	Lycopene	Total carotenoids	Ubiquinone	Ergosterol
CN 13 + AS	0.353±0.024	0.153±0.024	0.452±0.033	0.093±0.024	1.665±0.058	0.943±0.124	1.036±0.202
CN 13 + urea	0.505±0.064	0.505±0.068	0.038±0.006	0.376±0.047	1.545±0.023	1.728±0.163	3.484±0.633
CN 25 + AS	0.215±0.046	0.215±0.040	0.119±0.026	0.086±0.678	0.953±0.160	4.621±1.444	1.949±0.487
CN 25 + urea	0.366±0.027	0.166±0.036	0.411±0.001	0.013±0.002	1.228±0.007	1.414±0.320	1.342±0.315
CN 50 + AS	0.519±0.079	0.519±0.164	0.125±0.008	0.115±0.016	1.454±0.038	1.139±0.275	2.145±0.360
CN50 + urea	0.476±0.058	0.276±0.085	0.095±0.019	0.212±0.032	1.235±0.037	1.753±0.378	1.987±0.401

Table S3: HPLC analysis of screening cultivations of *S. roseus* cultivated on media containing SCG hydrolysate with different C/N ratio. Productions are listed in mg/g of cell dry weight.

Media type	Betacarotene	Torularhodin	Torulene	Lycopene	Total carotenoids	Ubiquinone	Ergosterol
CN 13 + AS	0.468±0.075	0.299±0.053	0.412±0.077	0.118±0.025	1.781±0.183	1.464±0.215	6.002±1.275
CN 13 + urea	0.579±0.08	0.485±0.114	0.418±0.081	0.013±0.003	1.697±0.266	2.151±0.677	6.387±0.936
CN 25 + AS	0.618±0.12	0.171±0.038	0.508±0.09	0.111±0.02	1.428±0.303	1.98±0.427	5.508±1.666
CN 25 + urea	0.614±0.108	0.213±0.043	0.417±0.093	0.094±0.012	1.913±0.299	1.812±0.463	2.852±0.839
CN 50 + AS	0.509±0.109	0.278±0.054	0.418±0.084	0.089±0.02	1.298±0.41	2.104±0.409	3.453±0.427
CN50 + urea	0.549±0.116	0.378±0.089	0.387±0.116	0.128±0.033	1.504±0.333	1.928±0.446	2.846±1.183

Table S4: HPLC analysis of screening cultivations of *S. salmonicolor* cultivated on media containing SCG hydrolysate with different C/N ratio. Productions are listed in mg/g of cell dry weight.

Media type	Betacarotene	Torularhodin	Torulene	Lycopene	Total carotenoids	Ubiquinone	Ergosterol
CN 13 + AS	0.651±0.107	0.253±0.055	0.411±0.076	0.07±0.0141	1.702±0.297	1.827±0.282	6.039±0.596
CN 13 + urea	0.559±0.121	0.136±0.030	0.321±0.053	0.009±0.002	2.334±0.405	0.572±0.122	2.643±0.571
CN 25 + AS	0.537±0.104	0.137±0.027	0.343±0.060	0.076±0.024	1.489±0.274	1.214±0.215	3.618±1.086
CN 25 + urea	0.487±0.092	0.127±0.026	0.488±0.076	0.035±0.007	1.909±0.411	0.846±0.205	2.958±0.575
CN 50 + AS	0.469±0.084	0.192±0.037	0.087±0.021	0.033±0.009	1.009±0.132	1.095±0.291	3.199±0.640
CN50 + urea	0.410±0.064	0.113±0.020	0.348±0.077	0.124±0.017	1.221±0.191	1.247±0.244	3.289±0.610

Table S5: HPLC analysis of *S. pararoseus*: 96 hours bioreactor cultivation on SCG hydrolysate + coffee oil. Productions are listed in mg/g of cell dry weight.

Cultivation time	Betacarotene	Torularhodin	Torulene	Lycopene	Total carotenoids	Ubiquinone	Ergosterol
0 h	1.232±0.204	0.124±0.024	0.284±0.061	0.105±0.023	1.982±0.445	4.826±0.989	5.816±1.306
18 h	1.453±0.211	0.632±0.199	3.264±0.510	0.240±0.048	3.820±1.069	5.249±1.039	6.534±1.299
24 h	1.718±0.438	0.826±0.178	3.198±0.687	0.328±0.060	6.741±1.796	6.318±0.850	6.472±1.434
44 h	3.102±0.936	0.742±0.107	2.546±0.396	0.173±0.023	6.982±2.201	7.982±2.925	13.849±2.020
48 h	2.988±0.662	1.126±0.243	1.844±0.359	0.326±0.070	7.217±0.937	8.317±2.067	13.261±2.857
68 h	3.116±0.840	1.324±0.297	1.247±0.165	0.242±0.041	7.416±1.179	8.023±1.697	13.983±1.818
72 h	3.016±0.641	0.982±0.162	0.901±0.131	0.265±0.044	6.921±1.369	8.316±0.955	13.686±1.527
96 h	2.918±0.336	0.741±0.093	0.741±0.099	0.185±0.033	5.816±1.312	8.265±1.648	13.485±1.275

Table S6: HPLC analysis of *R. toruloides*: 96 hours bioreactor cultivation on SCG hydrolysate + coffee oil. Productions are listed in mg/g of cell dry weight.

Cultivation time	Betacarotene	Torularhodin	Torulene	Lycopene	Total carotenoids	Ubiquinone	Ergosterol
0 h	0.562±0.096	1.574±0.246	0.108±0.078	1.033±0.288	3.957±0.458	3.527±0.795	3.635±0.563
24 h	0.175±0.046	1.548±0.256	0.211±0.026	1.151±0.412	3.734±0.735	2.741±0.418	3.692±0.996
48 h	0.337±0.082	3.749±0.425	0.336±0.083	1.006±0.246	5.543±1.385	1.829±0.337	5.249±1.181
72 h	0.446±0.094	5.961±1.107	0.382±0.063	1.039±0.239	8.283±1.023	3.713±1.172	6.045±1.206
96 h	0.866±0.173	7.704±1.037	0.511±0.098	1.005±0.254	10.02±1.568	3.710±0.961	6.440±1.433

Table S7: HPLC analysis of *S. pararoseus*: 96 hours bioreactor cultivation on SCG hydrolysate + waste frying oil. Productions are listed in mg/g of cell dry weight.

Cultivation time	Betacarotene	Torularhodin	Torulene	Lycopene	Total carotenoids	Ubiquinone	Ergosterol
0 h	0.028±0.006	-	0.084±0.011	-	0.117±0.037	1.710±0.430	0.781±0.137
16 h	0.045±0.005	0.310±0.057	0.092±0.015	0.241±0.027	0.731±0.158	1.332±0.295	2.074±0.454
22 h	0.008±0.003	0.095±0.015	0.073±0.024	0.328±0.067	0.527±0.133	0.870±0.174	0.811±0.190
40 h	0.133±0.018	0.551±0.117	0.153±0.026	0.173±0.027	1.155±0.175	6.087±0.955	2.457±0.545
48 h	0.050±0.011	0.780±0.103	0.204±0.076	-	1.104±0.170	7.047±0.958	2.861±0.565
60 h	0.095±0.031	2.822±0.447	0.155±0.034	-	3.393±0.766	3.046±0.657	4.673±1.297
70 h	0.109±0.013	3.302±0.602	0.089±0.019	-	3.539±0.764	4.287±0.914	3.921±0.853
72 h	0.071±0.014	1.833±0.432	0.232±0.046	-	2.404±0.508	7.294±1.140	3.947±0.853
90 h	0.045±0.010	2.066±0.251	0.18±0.0240	-	2.622±0.507	5.179±0.628	3.023±0.672
96 h	0.067±0.017	3.290±0.663	0.288±0.053	0.453±0.102	5.172±1.032	9.079±1.417	5.733±1.115

Table S8: HPLC analysis of *R. toruloides*: 96 hours bioreactor cultivation on SCG hydrolysate + waste frying oil. Productions are listed in mg/g of cell dry weight.

Cultivation time	Betacarotene	Torularhodin	Torulene	Lycopene	Total carotenoids	Ubiquinone	Ergosterol
0 h	0.102±0.013	2.457±0.483	0.069±0.023	-	2.651±0.445	5.698±1.121	5.356±0.673
24 h	0.168±0.028	1.243±0.268	0.121±0.031	-	1.803±0.261	4.091±0.663	4.949±0.777
40 h	0.202±0.050	1.002±0.126	0.102±0.013	-	1.302±0.344	4.034±0.544	2.406±0.606
48 h	0.048±0.011	3.143±0.743	0.049±0.011	-	3.268±0.691	4.224±0.904	4.755±0.744
54 h	0.059±0.012	2.589±0.434	0.155±0.024	-	2.827±0.667	3.537±0.818	5.791±1.109
56 h	0.228±0.046	2.112±0.383	0.128±0.025	0.386±0.045	2.870±0.619	5.803±1.164	2.322±0.259
72 h	0.166±0.032	2.365±0.311	0.095±0.025	-	2.641±0.526	3.001±0.584	5.071±1.174
78 h	0.026±0.004	4.277±0.965	0.117±0.016	0.345±0.053	4.766±0.708	4.285±0.951	4.181±0.653
96 h	0.114±0.024	2.662±0.789	0.067±0.008	-	2.866±0.758	2.226±0.472	3.705±0.984

Table S9: HPLC analysis of *S. pararoseus*: 96 hours bioreactor cultivation on SCG hydrolysate + waste animal fat. Productions are listed in mg/g of cell dry weight.

Cultivation time	Betacarotene	Torularhodin	Torulene	Lycopene	Total carotenoids	Ubiquinone	Ergosterol
18 h	1.539±0.686	-	0.5532±0.100	0.006±0.009	3.402±0.178	3.081±0.903	5.147±0.852
24 h	2.176±1.023	1.006±0.330	0.615±0.232	0.313±0.098	4.213±0.311	4.931±0.871	7.964±0.478
26 h	2.129±0.977	0.533±0.178	1.055±0.408	0.322±0.141	3.405±0.402	5.950±1,203	4.240±0.963
44h	2.046±1.232	1.711±0.435	1.568±0.646	0.608±0.208	4.751±0.378	7.793±1,087	8.2±0.1021
52 h	3.367±1.415	0.914±0.378	1.054±0.317	0.521±0.115	5.327±0.364	6.211±0.843	8.225±0.577
54 h	3.268±0.968	1.041±0.356	1.065±0.511	0.323±0.108	6.721±0.470	5.051±0.479	10.62±0.637
72 h	3.046±1.108	1.171±0.408	1.226±0.647	0.909±0.206	7.292±0.672	6.362±0.607	9.365±0.804
96 h	4.506±0.976	1.409±0.311	-	1.364±0.314	8.745±0.819	7.085±0.780	10.817±1.012

Table S10: HPLC analysis of *R. toruloides*: 96 hours bioreactor cultivation on SCG hydrolysate + waste animal fat. Productions are listed in mg/g of cell dry weight.

Cultivation time	Betacarotene	Torularhodin	Torulene	Lycopene	Total carotenoids	Ubiquinone	Ergosterol
2 h	2.007±0.101	0.648±0.102	0.119±0.066	0.529±0.100	3.410±0.867	8.617±2.001	3.931±1.006
08 h	0.781±0.230	0.708±0.115	0.075±0.033	0.472±0.099	2.207±0.620	7.429±1.250	4.026±1.322
24 h	1.697±0.187	0.709±0.208	0.522±0.087	0.136±0.055	3.448±1.002	6.774±1.645	4.145±0.871
48 h	1.753±0.345	1.228±0.316	0.441±0.103	0.115±0.040	3.891±0.983	7.211±1.531	5.766±0.838
72 h	2.062±0.726	0.502±0.185	0.802±0.241	0.136±0.047	3.981±1.311	7.853±1.050	6.602±1.430
78 h	1.899±0.442	0.156±0.075	0.924±0.178	0.102±0.032	3.946±1.415	7.105±2.130	8.853±1.308
96 h	1.778±0.628	0.131±0.064	0.900±0.196	0.081±0.027	3.318±0.977	7.674±1.311	7.296±1.131

Table S11: HPLC analysis of phenolic content in different cultivation media according to the C/N ratio at the beginning of the cultivation. Productions are listed in mg/L of media.

Cultivation media	C/N 13	C/N 25	C/N 50	Bioreactor C/N 25
Total phenolics	2.672±0.178	5.031±0.318	8.345±0.247	4.894±0.316
Gallic acid	0.238±0.074	0.647±0.130	1.204±0.412	0.871±0.282
Chlorgenic acid	1.871±0,241	3.602±0,208	6.831±0,441	3.715±0,198
Caffeis acid	0.008±0.003	0.020±0.008	0.038±0.011	0.026±0.012
Quercetin-3-glucoside	0.047±0.008	0.093±0.028	0.173±0.042	0.117±0.034
Kaempferol-3-glucoside	0.052±0.011	0.124±0.031	0.276±0.084	0.127±0.043
Quercetin	0.048±0.012	0.102±0.021	0.174±0.041	0.134±0.027
Kaempferol	0.028±0.010	0.064±0.019	0.124±0.030	0.059±0.022
